# On the Characteristics of Fatigue Fracture with Rapid Frequency Change

**DOI:** 10.3390/e25060840

**Published:** 2023-05-24

**Authors:** Mohammad A. Amooie, K. P. Lijesh, Ali Mahmoudi, Elaheh Azizian-Farsani, Michael M. Khonsari

**Affiliations:** Department of Mechanical and Industrial Engineering, Louisiana State University, Baton Rouge, LA 70803, USAlijesh_mech@yahoo.co.in (K.P.L.);

**Keywords:** fracture fatigue entropy (FFE), frequency dependence, internal friction, unstable working conditions, fatigue life

## Abstract

The effect of sudden variations in working modes and fatigue behavior of CS 1018 is studied. A general model based on the framework of the fracture fatigue entropy (FFE) concept is developed to capture such changes. Fully reversed bending tests are performed on flat dog bone specimens with a series of variable frequency tests without turning the machine off to simulate fluctuating working conditions. The results are then post-processed and analyzed to assess how fatigue life changes when a component is subjected to sudden changes in multiple frequencies. It is demonstrated that regardless of the frequency changes, FFE remains constant and stays within a narrow band range, similar to that of a constant frequency.

## 1. Introduction

Fatigue is the most dangerous type of component failure subjected to cyclic loading. While the existing literature is rich with volumes of research devoted to the prediction of fatigue life under constant operating conditions, far less attention has been given to practical situations in which the component experiences sudden changes in the operating conditions, such as the working frequency. This is the focus of the present study.

We begin by providing a brief review of pertinent publications. Resis et al. [1] studied the effect of a change in frequency on the fatigue life of epoxy–steel lap joints for frequencies between 2 and 40 Hz. The results were verified using the S-N curve for a constant frequency and linear cumulative damage rule, introduced in Equation (1).

(1) $$\sum \frac{n_{i}}{N_{i}}=1$$

where $n_{i}$ is defined as the number of cycles running at a specific condition, e.g., load or frequency, and $N_{i}$ is the corresponding number of cycles to failure at each operating load or frequency. Resis et al. [1] showed that the life dependency on frequency is subject to change with shear stress level, meaning that fatigue life experiences a more appreciable change at lower stress levels. More specifically, they reported that fatigue life increases from low frequencies up to 10 Hz, but tends to decrease at frequencies higher than 10 Hz.

Zhu et al. [2] investigated the effect of various environmental conditions and frequencies on the fatigue life of E319 cast aluminum alloy. They conducted multiple tests at different loading frequencies in different environments, such as air, water, and water vapor. The results showed an increase in fatigue strength at high frequencies. Tsutsumi et al. [3] investigated the influence of testing frequency on low-carbon steel fatigue life. The results showed an increase in the fatigue life of the specimen subjected to frequencies in the range of 20 kHz compared to those tested at 10 Hz. In addition, their results revealed that, due to thermal activation of screw dislocations, the stress level increased by an increase in strain rate in split Hopkinson bar test and yielded a denser presence of slip bands in the vicinity of crack tip for low-frequency tests compared to those at higher tested frequencies.

Advances in ultrasonic fatigue testing machines facilitated high-frequency cyclic loading [4,5] and research results revealed that frequency can significantly affect fatigue life [6]. Zhu et al. [7] investigated the effect of frequency on the fatigue life of high-cycle, low-strength Cr-Ni-Mo-V steel welded joints. They concluded that fatigue strength increases at ultrasonic frequencies. In 2016, Schneider et al. [8] investigated the effect of frequency on the fatigue behavior of 50CrMo4 and EN AW-5083. Tests were conducted with an ordinary fatigue tester with frequencies below 400 Hz and an ultrasonic fatigue tester capable of running cyclic loads with 20 kHz frequency. The results depicted a dependency of the frequency on the fatigue life of tested materials. Such variations were attributed to different parameters such as temperature, environment, and strain rate.

In 2023, Tahmasbi et al. [9] published a comprehensive review analyzing the correlation between fatigue life and frequency. The article summarizes that increasing loading frequency results in an increase in internal friction and leads to a reduction in fatigue strength. The authors contend that macro crack growth can be considered as an independent parameter from the loading frequency. They suggest that the impact of frequency on SN curves for loading frequencies between 10 and 100 Hz is small; however, this impact shows itself more significantly when the part is working in corrosive or variable temperature environments. Importantly, materials with BCC structures are subject to more significant changes in life with changes in frequency. Yet, a general framework that explains such variations appears to be lacking. More specifically, most studies analyze steady conditions and do not account for sudden changes in stress amplitude or frequency alterations.

Komorek et al. [10] investigated the temperature dependency of epoxy resin-based polymers on the frequency of loading. The results showed that with increasing the frequency, the surface temperature of the tested material increases. Deutscher et al. [11] concluded that the maximum stress level, frequency, and maximum grain size of concrete has a critical impact on the temperature rise of the sample in fatigue testing process. Xiao and Hamouz [12] reported an increase in the heat generation rate with an increase in frequency of angle-ply AS4/PEEK. Klusak et al. [13] showed an increase in frequency leads to an increase in the temperature of S355J0 and S355J2 during fatigue degradation process.

Recently, the principles of thermodynamics have been applied to examine fatigue degradation [14,15]. One such method that takes advantage of the laws of irreversible thermodynamics is fracture fatigue entropy (FFE), introduced by Naderi et al. [16] in 2010. FFE is the accumulated generated entropy until the fracture of a material. Research shows that FFE is a constant value for a specific material; does not depend on frequency, stress amplitude, geometry, or testing conditions [17,18,19]; and can be viewed as a material property. An extensive literature review shows that the FFE approach can provide reliable information on the fatigue degradation of different materials, including metals and composites, under different loading conditions [20,21,22,23,24,25,26,27,28,29,30,31,32,33,34,35,36]. What is lacking is pertinent information on the fatigue characteristics of components that experience sudden changes in frequency.

In the present study, we investigate the application of fracture fatigue entropy in fatigue cases subjected to sudden frequency changes. To evaluate the efficacy of the method, extensive sets of experiments are carried out and the results are presented in this paper. The method covers different variable frequencies: low–high, high–low, low–high–low, and high–low–high. The accumulated entropy values up to fracture are presented and compared to provide insight into the application of the FFE method in components experiencing variable frequency.

The outline of this paper is as follows. Section 2 introduces a mathematical model based on the law of thermodynamics that extends the formulation to multiple frequency changes. Section 3 describes material specification and preparation, and Section 4 points out the testing procedure. Section 5 and Section 6 present the results and the applications of the introduced method, respectively. Section 7 summarizes the key findings of the paper.

## 2. Theory and Formulation

**a.**  **First and Second Laws of Thermodynamics**

Fatigue degradation is an irreversible thermodynamic process that generates heat in the material. Therefore, a thermodynamic framework can be used to formulate the fatigue process. For a material undergoing fatigue with small deformations, the law of conservation of energy (the 1st law of thermodynamics) for an arbitrary control volume can be written as [37]:

(2) $$\rho \dot{e}=\sigma :\dot{\varepsilon }-div\vec{q}$$

where ρ denotes density and e is the change in specific internal energy. σ is the applied stress and ε is the total strain. $\sigma :\dot{\varepsilon }$ represents the mechanical energy generation in the material. $\vec{q}$ is the heat flux across the boundaries of a control volume. According to the Clausius–Duhem inequality (the 2nd law of thermodynamics), the change in entropy of a system is always greater than or equal to the rate of heat flux divided by temperature [37].

(3) $$\rho \frac{ds}{dt}\geq -div\frac{\vec{q}}{T}$$

where s is the specific entropy (i.e., per unit volume), t denotes time, and T represents temperature in the Kelvin scale. To relate the first and second laws of thermodynamics, Helmholtz free energy is utilized as:

(4) ψ=e−Ts

Using Helmholtz’s free energy function, ψ, and after some manipulations, which are presented in detail in references [16,37], the Clasius–Duhem inequality written as follows in terms of the entropy generation, ${\dot{S}}_{g}$. can be written as:

(5) $${\dot{S}}_{g}=\frac{\sigma :{\dot{\varepsilon }}^{p}}{T}+\frac{A\dot{V}}{T}-\frac{\vec{q}.\nabla T}{T^{2}}\geq 0$$

It states that the rate of non-negative generated entropy, ${\dot{S}}_{g}$, is due to three different mechanisms: plastic strain energy dissipation ($\sigma :{\dot{\varepsilon }}^{p}$), nonrecoverable stored energy in material ($A\dot{V}$), and the heat dissipation owing to heat conduction ($\vec{q}.\nabla T/T$). Research shows that the dominant mechanism of entropy generation in metals is the generated heat owed to plastic strain energy (${\dot{W}}_{p}=\sigma :{\dot{\varepsilon }}^{p}$) [38]. Therefore, the accumulated entropy up to failure can be calculated as:

(6) $$S_{f}=\int_{0}^{t_{f}}\frac{{\dot{W}}_{p}}{T}dt$$

where $S_{f}$ and $t_{f}$ are the accumulated entropy and time to fracture, respectively. It shows that fracture entropy can be evaluated if the energy generation owing to plastic deformation is determined.

**b.**  **Internal heat generation**

**Figure 1:**
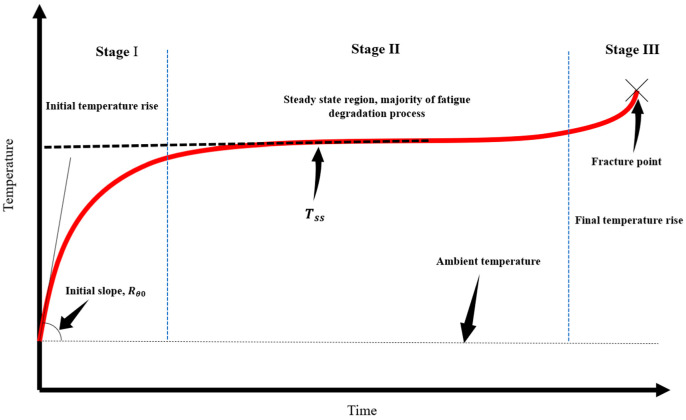
Temperature evolution during cyclic loading and presentation of the initial temperature slope.

Among different approaches, it has been shown that thermography offers many advantages for quantifying the internal heat generation and plastic strain energy in materials undergoing fatigue. Advancements in infrared cameras have made it possible for researchers to measure surface temperature precisely and use this information to assess fatigue damage [39,40]. This is directly proportional to fatigue load and actuation frequency [6,41,42]. The higher the stress or the actuation frequency of loading, the greater is the increase in surface temperature. As shown in Figure 1, the temperature of a component undergoing fatigue goes through three stages before it breaks. In the first stage, there is a quick increase in temperature when the cyclic loading begins. Then there is a stabilization stage (steady state) where the temperature remains constant. Finally, there is a sudden increase in temperature just before fracture in the third phase [43]. In this section, two thermography test cases are discussed, and formulations are presented to evaluate the internal heat generation.

***Slope of Temperature Rise***

The slope of temperature rise at the beginning of the fatigue process can serve as a measure of internal heat generation. The details of formulations are presented in references [19,44]. Here, the key features are presented. The law of conservation of energy for a component undergoing fatigue can be written as [37]:

(7) $$\Delta U={\dot{Q}}_{in}+{\dot{Q}}_{gen}-{\dot{Q}}_{out}$$

where ΔU is the rate of internal energy change, ${\dot{Q}}_{in}$ represents the energy that enters the control volume, ${\dot{Q}}_{gen}$ is the energy generated in the material, and ${\dot{Q}}_{out}$ is the energy that dissipates to the surrounding through conduction, convection, and radiation. The state of internal energy is directly proportional to the temperature; hence, ΔU can be replaced by:

(8) $$\Delta U=\rho c_{p}\frac{dT}{dt}$$

where ρ is density, $c_{p}$ denotes the specific heat capacity, and T represents temperature. Considering that ${\dot{Q}}_{out}$ can be dissipated to the environment through conduction, convection, and radiation. It can be assumed that ${\dot{Q}}_{in}=0$ in Equation (7) because no heat enters the control volume. In addition, at the beginning of the fatigue process, there is no temperature gradient between the component and the environment and there is no time to dissipate energy to the surrounding. Therefore, ${\dot{Q}}_{out}$ is negligible.

Letting $R_{\theta }=dT/dt$, Equation (7) can be rewritten as:

(9) $${\dot{Q}}_{gen}=\rho c_{p}R_{\theta }$$

Equation (9) states that the internal energy generation can be estimated using the slope of temperature rise at the beginning of the fatigue process.

***Slope of Cooling Curve***

Meneghetti [41] showed that internal heat generation can be obtained by measuring the slope of the temperature drop after stopping the fatigue test at the steady-state phase. At the steady-state phase, the temperature is nearly constant. Therefore, the change in internal energy is negligible, ΔU≅0 for the time just before interrupting the test, $t=t_{i}^{-}$. As no energy enters the control volume, ${\dot{Q}}_{in}=0$, the first law of thermodynamic in Equation (7) can be written as:

(10) $${\dot{Q}}_{gen}={\dot{Q}}_{out}\;\;\;\;at\;t=t_{i}^{-}$$

Equation (10) states that at steady state, the heat generated in material and heat dissipated to the environment are in equilibrium. Upon halting the experiment at $t=t_{i}^{+}$, there will be no heat generation in material, and Equation (7) converts to:

(11) $$\rho c_{p}\frac{dT}{dt}={-\dot{Q}}_{out}\;\;\;at\;t=t_{i}^{+}$$

Equation (12) shows that the rate of heat dissipation can be estimated using the slope of temperature drop just after stopping the fatigue test. From $t=t_{i}^{-}$ to $=t_{i}^{+}$, the change in temperature is negligible, so ${\dot{Q}}_{out}$ remains nearly constant. Replacing ${\dot{Q}}_{out}$ from Equations (10) in (11), and changing dT/dt with $R_{\theta }$, the internal heat generation can be obtained as:

(12) $${\dot{Q}}_{gen}=-\rho c_{p}R_{\theta }$$

It shows that the generated energy can be estimated using the rate of temperature drop by stopping the fatigue test at the steady-state phase. It is noted that the $R_{\theta }$ has a negative value in Equation (12) due to the cooling effect. Jang and Khonsari [19] analytically showed that the rate of temperature rise at the beginning of the fatigue process is identical to the rate of temperature drop at the steady states if the internal heat generation remains constant throughout fatigue.

**c.**  **Generalization of governing equations for variable frequency**

**Figure 2:**
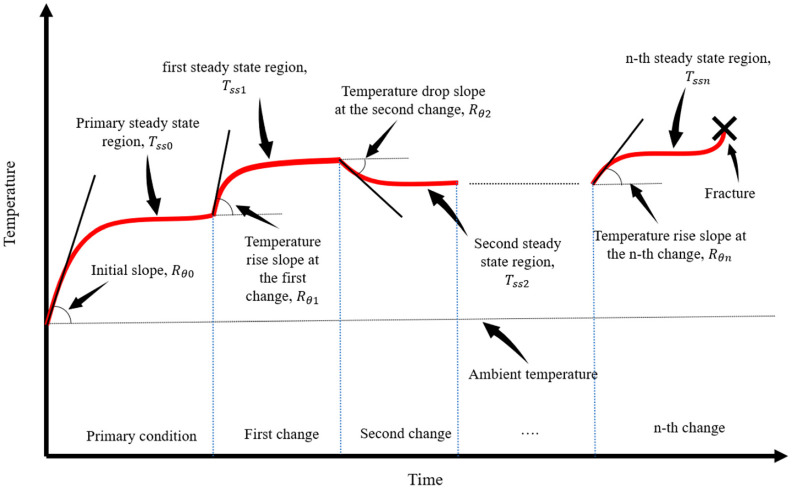
Temperature variations with an arbitrary change in working frequency.

Internal heat generation during fatigue is directly related to the frequency of loading. The higher the frequency, the larger is the temperature rise. The accumulated entropy at a constant frequency can be obtained from the following equation [19].

(13) $$S_{f}=\frac{N_{f}{\dot{Q}}_{gen}}{T_{ss}f}$$

where $N_{f}$ is the number of cycles to failure, f is the loading frequency, and $T_{ss}$ is the temperature of the steady state. ${\dot{Q}}_{gen}$ internal heat generation owing to damaging mechanisms such as plastic strain energy (${\dot{W}}_{p}$) in metals. If a multi-step load with n load blocks (Figure 2) is applied to a component, the accumulated entropy up to fracture can be obtained as:

(14) $$S_{f}=\sum_{i=1}^{n}\frac{N_{i}{\dot{Q}}_{gen,i}}{T_{ss,i}f_{i}}$$

where $N_{i}$ and ${\dot{Q}}_{gen,i}$ are the number of fatigue cycles and the internal damaging heat generation at ith load step, respectively. $T_{ss,i}$ represents the steady-state temperature and $f_{i}$ is the frequency of load step i.

Referring to Figure 2, upon changing the frequency at $=t_{i}$, the temperature settles into a new steady state. If the second frequency is higher than the first, the temperature increases. Otherwise, the temperature drops if the second frequency is lower than the initial frequency. At $t=t_{i}^{-}$, just before changing the frequency, Equation (7) can be written as:

(15) $${\dot{Q}}_{out}={\dot{Q}}_{gen,0}=\rho c_{p}{R_{\theta }}_{0}\;\;\;at\;t=t_{i}^{-}$$

where ${R_{\theta }}_{0}$ is the initial slope of temperature at the beginning of the fatigue process and ${\dot{Q}}_{gen,0}$ is the rate of internal heat generation in the initial step before changing the frequency. Upon changing the frequency at $t=t_{i}^{+}$. Replacing ΔU with $\rho c_{p}{R_{\theta }}_{1}$ in Equation (7) yields

(16) $$\rho c_{p}{R_{\theta }}_{1}={\dot{Q}}_{gen,1}-{\dot{Q}}_{out}\;\;\;at\;t=t_{i}^{+}$$

where ${R_{\theta }}_{1}$ is the rate of temperature change just after frequency changes. ${\dot{Q}}_{gen,1}$ is the rate of internal heat generation at the second frequency. As there is not enough time from $t=t_{i}^{-}$ to $t=t_{i}^{+}$ for the temperature to change, ${\dot{Q}}_{out}$ remains constant. Replacing ${\dot{Q}}_{out}$ from Equation (15) into Equation (16) leads to:

(17) $${\dot{Q}}_{gen,1}=\rho c_{p}({R_{\theta }}_{0}+{R_{\theta }}_{1})$$

Equation (17) states that the rate of energy production after frequency change can be obtained using the initial slope of temperature and the slope of temperature after changing the loading frequency. Now, if a multi-step load as shown in Figure 2, is applied to a fatigue component, the energy generation in each step can be obtained using the extended version of Equation (17):

(18) $${\dot{Q}}_{gen,n}=\rho c_{p}\sum_{i=0}^{n}\left({R_{\theta }}_{i}\right)$$

where ${\dot{Q}}_{gen,n}$ is the rate of internal heat generation in step n and ${R_{\theta }}_{i}$ is the rate of temperature change at the beginning of step i. Using Equation (14) for accumulated entropy for a multi-step load and replacing ${\dot{Q}}_{gen,i}$ with ${\dot{Q}}_{gen,n}$ from Equation (18), the total entropy generation up to fracture for a variable frequency loading can be obtained as:

(19) $$S_{f}=\rho c_{p}\sum_{i=0}^{n}\frac{N_{i}}{T_{ss,i}f_{i}}\sum_{j=0}^{i}\left({R_{\theta }}_{j}\right)$$

**d.**  **Internal Friction and Fatigue Limit**

Fatigue process involves a combination of plastic, elastic, and anelastic deformation. Internal friction is a non-damaging phenomenon resulting from inherent flaws in the form of half-planes that can sway within the material’s lattice during repeated loading. Such planes exist inside the material’s matrix due to defects that are generated during the manufacturing process. Under cyclic loading, the applied stress provides sufficient activation energy or enthalpy for these half-planes to experience edge dislocations. This occurs when lines of atoms in these half-planes transition from one energy level or Peierls valley to another. The transition of these kink bands gradually returns them to their initial positions, resulting in no damaging effects. Hence, materials subjected to stress levels below the fatigue limit can undergo a high number of cycles without experiencing damage. This non-damaging and recoverable concept contributes to the initial slope of temperature rise during the fatigue process. The interested reader in the mechanism and the source of internal friction is referred to reference [18,45,46]. The effect of internal friction should be considered in determining the total accumulated entropy until fracture. The experimental findings indicate a linear relationship between the non-damaging term and the damaging part, demonstrating their interdependence.

**Figure 3:**
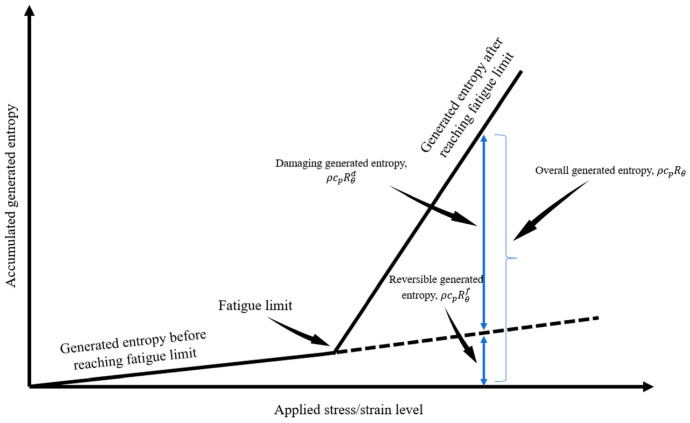
The role of internal friction in temperature evolution slope.

Figure 3 provides a schematic view of how internal friction plays a role in generated entropy during the fatigue degradation process. The effect of this non-damaging energy for stresses above the endurance limit must be accounted for in calculating the total accumulated entropy [47].

As shown in Figure 3, internal friction contributes to the slope of temperature evolution, and this contribution is considered a non-damaging source of heat generation [48]. Therefore, Equation (20) can be written to express the role of internal friction [18].

(20) $$R_{\theta }=R_{\theta }^{d}+R_{\theta }^{f}$$

Thus, the damaging entropy generation can be evaluated from the following equation.

(21) $$S_{f}=\rho c_{p}\sum_{i=0}^{n}\frac{N_{i}}{T_{ss,i}f_{i}}\sum_{j=0}^{i}\left({{(R}_{\theta }-R_{\theta }^{f})}_{j}\right)$$

## 3. Material and Specifications

**Figure 4:**
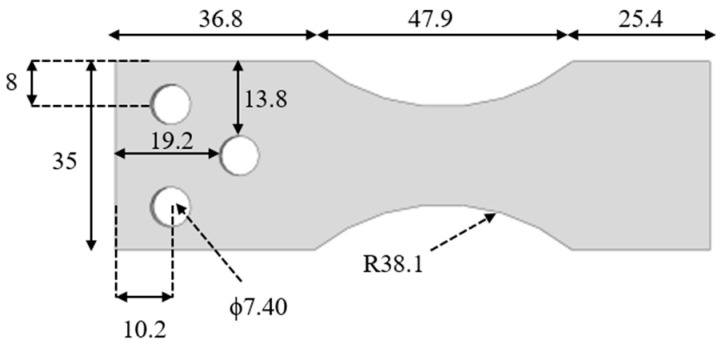
Schematic figure of the bending specimen and the dimensions (all in mm).

The material used for the experimental procedure is Carbon Steel 1018 flat dog bone specimens (Figure 4) which are designed particularly for fully reversed bending tests. Two circular arcs with a radius of 38.1 mm provide stress concentration to better study the fracture area. Specimens are designed based on the American Society for Testing and Material Special Technical Publication 566 (ASTM STP 566) standard [49]. The thickness of specimens is 3.01 mm, and all specimens are polished on both surfaces up to 0.2 µm to reduce the impact of micro cracks on crack initiation and propagation on the surface. Material chemical composition and mechanical properties are provided in Table 1 and Table 2, respectively. One side of the surface is painted black to increase the emissivity near to 1 for IR camera data acquisition purposes.

## 4. Bending Test Rig, Sensors, and Experimental Process

**Figure 5:**
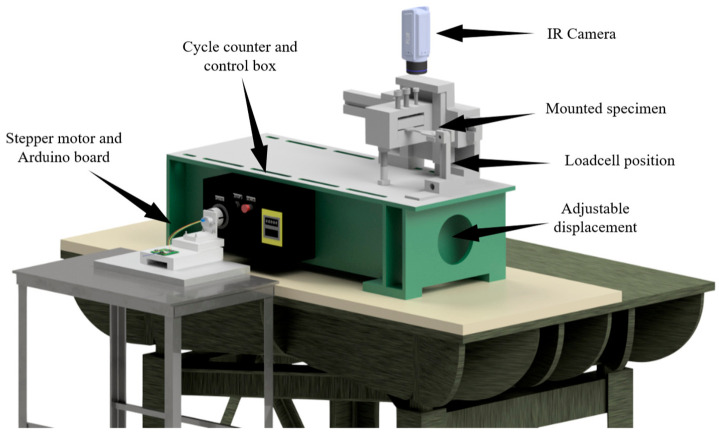
Schematic of the bending tester, IR camera, and stepper motor.

The testing apparatus is a fully reversed bending tester manufactured by Fatigue Dynamics, Inc. The fatigue machine is displacement adjustable, enabling the operator to adjust the displacement up to 25.4 mm. The machine also has a control box for adjusting the frequency and recording the number of cycles to failure. The operator can also change the frequency manually while the test is in progress. Moreover, a stepper motor programmed with an Arduino board was integrated into the control system to let the operator adjust the testing frequency precisely. An infrared camera (FLIR A600 series) is mounted on top of the specimen to record the temperature variations during the testing period (Figure 5). The camera has the capability of capturing data at up to 200 fps and at a resolution of 640 × 480 pixels and has the ability to record temperature changes as small as 50 mK with an accuracy of ±2 °C or ±2% for the reading. A modified actuating handle is designed to hold a load sensor between the actuation cam and the specimen to measure the load associated with a displacement (Figure 5), and the measured load is then converted to bending stress. A robust fatigue load cell (FUTEK LCF300) along with an analog voltage amplifier (FUTEK IAA100) and a DAQ (NI USB-6009) are used to record the maximum load applied on the specimen.

The testing process is planned in a way to make it possible to study a variety of operating frequencies. Reference frequencies of 30 to 24 Hz are chosen as the high frequencies, and working frequencies between 8 to 10 Hz are considered the low frequencies. First, constant frequency tests are conducted for 5.08 mm, 7.62 mm, and 10.16 mm displacements and the temperature evolutions are recorded and the FFEs are measured. For the next step, the 10.16 mm (345 MPa), 7.62 mm (316 MPa), and 5.08 mm (254 MPa) displacements (stress levels) are chosen for tests, and the frequency of tests are manipulated without letting the specimen experience any resting time and while the cyclic loading is imposed. The specimen is given time to reach a steady state before applying further changes. Numerous tests are conducted with random changes in frequency such as low–high, high–low, low–high–low, and high–low–high. The life and temperature evolution and variations for each testing condition are recorded. Two additional tests are conducted at 3.81 mm displacement, which corresponds to 211 MPa stress level. The two tests are conducted at high frequency and low–high frequencies to verify the functionality of the model for high-cycle fatigue tests. For the internal friction process, multiple tests are conducted from 1.27 mm (80 MPa) to 3.81 (211 MPa) mm with 2.54 mm intervals for 30 Hz and 10 Hz frequencies to investigate the impact of frequency on variations of internal friction. It is worth mentioning that the environmental conditions such as temperature have been monitored and maintained constant for all conducted tests.

## 5. Results and Discussion

**Figure 6:**
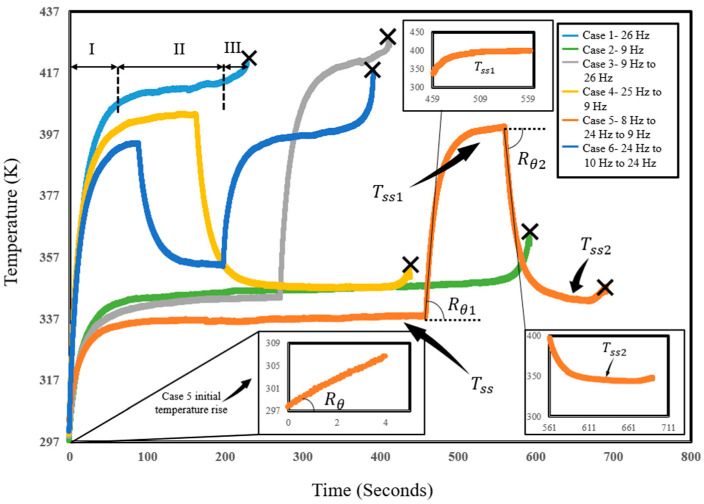
Temperature evolution profiles for 10.16 mm displacement at constant frequencies and arbitrary frequency variations during the test process.

The temperature evolution profiles for 10.16 mm tests with random changes in frequency are presented in Figure 6. Constant frequency (26 Hz) test temperature evolution is shown as reference, showing the three-phase evolution of temperature before fracture. Also shown is the temperature evolution for several cases that experience sudden changes in frequency. It should be noted that in all experiments the specimen reached a steady-state temperature before we applied a change in frequency. As the frequency drops or increases, the rates of plastic work and dislocation generation tend to change, bringing about a change in the temperature. Figure 6 also presents the rate of change at the beginning of each frequency change and the new steady-state temperature, which helps calculate the entropy generation rate for each stage.

**Figure 7:**
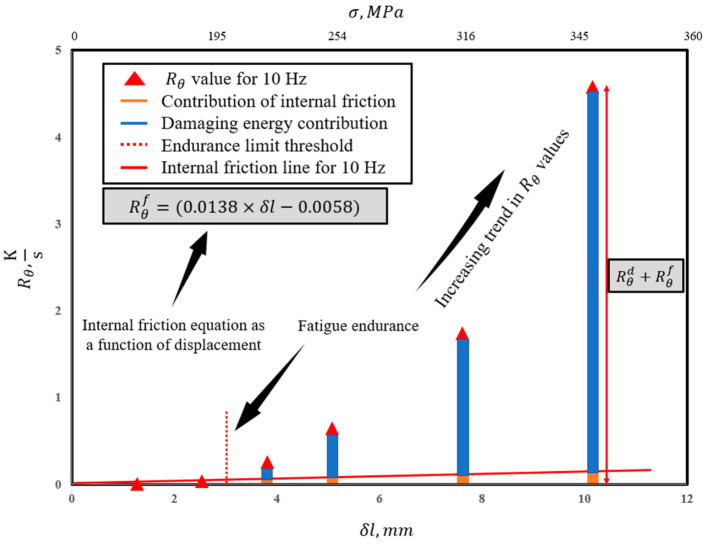
Internal friction line equation at 10 Hz.

**Figure 8:**
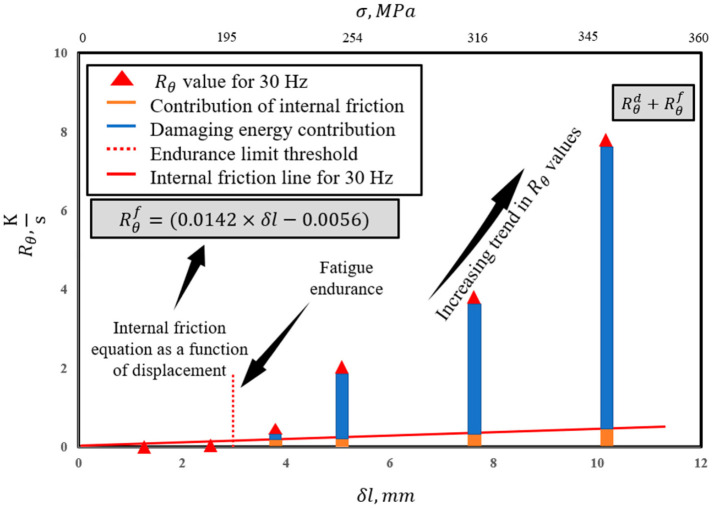
Internal friction line equation at 30 Hz.

Figure 7 and Figure 8 present the equations obtained for internal friction at 30 and 10 Hz, respectively (refer to Figure 3). The equations show very similar slopes for both frequencies; however, a slightly larger slope at 30 Hz is observed. This is attributed to the energy rate given to the system that creates a larger activation energy for the atoms in half-plane defects that tend to travel to their neighboring energy levels in the lattice with respect to the burger’s vector. For the simplicity of calculations, the equation for 30 Hz is assumed to apply to higher frequencies and the equation for 10 Hz is used for all lower frequencies.

**Figure 9:**
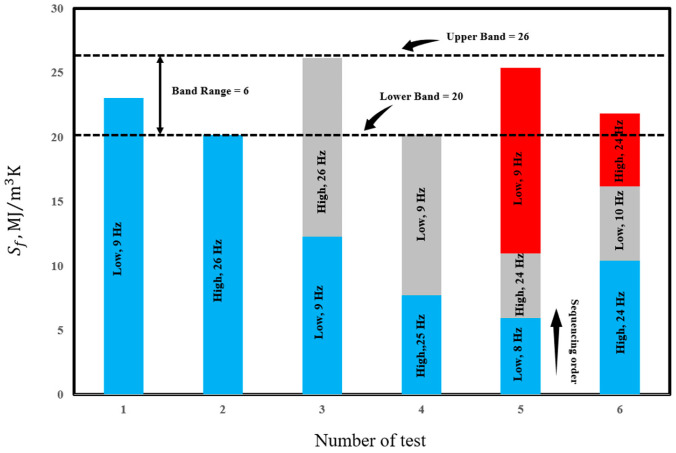
Calculated accumulated entropy generation for 10.16 mm under various frequency working conditions.

Figure 9 represents the entropy generation for each stage of the test at 10.16 mm (345 MPa) and the total entropy generation until the final failure occurs ($S_{f}$). Calculations for each stage are performed by deducting the energy generation due to internal friction for each stage considering the reference lines for high and low frequencies’ internal friction. As can be seen the total generated entropy remains nearly constant for all tested cases with an average value of 23 ± 3 MJm^−3^ K^−1^.

**Figure 10:**
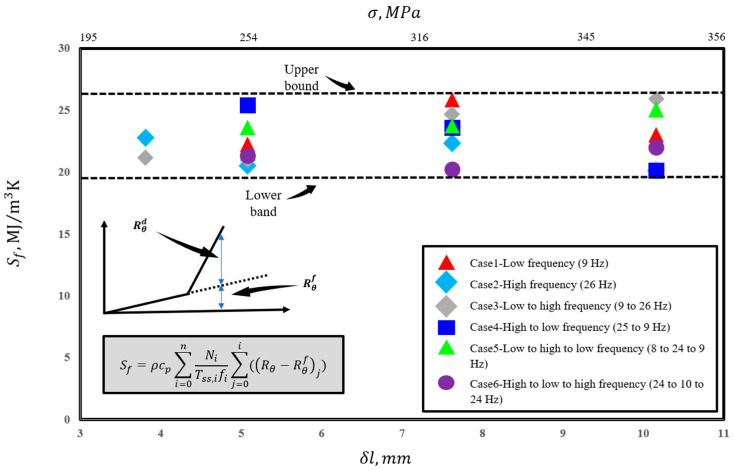
Total accumulated entropy for tested displacements.

The total accumulated entropy until failure for all tested displacements is presented in Figure 10. As it can be seen, the total generated entropy remains constant (~23 MJm^−3^ K^−1^) in a narrow band range (±3 MJm^−3^ K^−1^) for all tested displacements and frequency conditions. It should be noted that the values reported for each testing condition are based on taking the role of internal friction into account and deducting the values associated with non-damaging internal friction.

Table 3 summarizes the change in fatigue life for each displacement working and sudden frequency changes. The improvements in life for each condition with respect to the low-frequency test are reported. As it can be seen, low-frequency tests have the lowest life among all tested conditions. Similar observations have been reported in [50,51]. Reference [52] also reports lower fatigue life when a component operates at low frequency for environmental temperature. The report also states that the crack initiation mechanism is not dependent on the frequency [2]. This behavior can be attributed to the fact that for low-frequency tests, the material experiences longer periods of time under loading that led to the development of larger and more damaging cracks. In contrast, at higher frequencies, the specimen does not stay at the maximum stress level for a long period of time. Additionally, at low frequencies, the stress on the material may not be evenly distributed, causing stress concentrations that accelerate crack growth. Thus, at low frequencies, the slower rate of loading allows more time for the stress to accumulate and concentrate around geometric irregularities such as micro cracks on the surface or when the crack has formed. As a result, the stress distribution within the material becomes uneven, with higher-stress concentrations at these localized areas [3]. This change becomes more pronounced when the stress level imposed becomes larger (the maximum recorded difference is 5.55%). This can also be studied by investigating equation 19 for a case in which frequency sequencing does not occur. For a higher frequency, the rate of plastic work increases; however, an increase in frequency and steady-state temperature compensates for the increase in life and the plastic work rate. Table 3 also reveals that fatigue life improves when the specimen experiences frequency changes. The reason behind this change could be the accumulation of damage. When the material is subjected to constant frequency, the same areas of the material are repeatedly stressed at the same frequency; however, in variable frequency conditions, loading can distribute the stress throughout the material more evenly. The change in loading frequency might activate new planes in the material structural lattice to dislocate which means dynamic energy can be absorbed by new areas in the material. This leads to a more uniform distribution of damage instead of localized progressive damage, which can increase the life compared to constant frequency. The table also reveals that adding a frequency change improves life for tested frequencies. The reason may be due to the accumulation of damage as mentioned above. Nevertheless, the total generated entropy up to fracture, i.e., FFE, does not show any dependence on frequency sequencing.

## 6. Validation and Application

To investigate the utility of the model, two tests were conducted at 6.35 mm displacement (293 MPa stress level) with two frequency conditions. The first test is conducted at a 24 Hz constant frequency and the life is calculated using the accumulated entropy for fracture FFE = 23 MJm^−3^ K^−1^. The next test starts at 24 Hz, and then the frequency is dropped to 10 Hz after 12,300 cycles without turning the machine off. The remaining life is then calculated, and the prediction is compared with the result of the experiment. The internal friction lines are used to deduct the effect of non-damaging heat generation. Therefore, for the first test, we have:

Rθf@24Hz=0.0142×δl−0.0056→Rθf@24Hz=0.0142×6.35−0.0056=0.084Ks

Now having the initial slope for temperature evolution, we can write:

$$S_{f}=\rho c_{p}\sum_{i=0}^{0}\frac{N_{i}}{T_{ss,i}f_{i}}\sum_{j=0}^{0}\left({{(R}_{\theta }-R_{\theta }^{f})}_{j}\right)$$

The predicted number of cycles to failure can then be calculated as follows.

$$N_{f}=\frac{S_{f}\times T_{ss0}\times f}{\rho c_{p}(R_{\theta 0}-R_{\theta 0}^{f})}=\frac{23\times 373\times 24}{7.86\times 0.486\times (2.57-0.084)}=21,681$$

To verify the result, experiments were performed at the mentioned condition and the outcome shows that the number of cycles to failure is 22,070, i.e., with an error of 1.79%.

For the second case, the role of internal friction for 10 Hz frequency is calculated:

Rθf@10Hz=0.0138×δl−0.0058→Rθf@10Hz=0.0138×6.35−0.0058=0.081Ks

We let the specimen experience 12,300 cycles before changing the frequency to 10 Hz. We have:

$$S_{f}=\rho c_{p}\sum_{i=0}^{1}\frac{N_{i}}{T_{ss,i}f_{i}}\sum_{j=0}^{1}\left({{(R}_{\theta }-R_{\theta }^{f})}_{j}\right)\to S_{f}=\rho c_{p}(\frac{N_{0}}{T_{ss0}f_{0}}(R_{\theta 0}-R_{\theta 0}^{f})+\frac{N_{1}}{T_{ss1}f_{1}}((\sum_{i=0}^{1}R_{\theta })-R_{\theta 1}^{f})\;\;\;\;\;\;\;N_{1}=\frac{{(S}_{f}-S_{0})\times T_{ss1}\times f_{1}}{\rho c_{p}((\sum_{0}^{1}R_{\theta })-R_{\theta 1}^{f})}$$

$S_{f}=23\;and\;S_{0}$ can be calculated as follows:

$$S_{0}=\rho c_{p}\frac{N_{0}}{T_{ss0}f_{0}}\left(R_{\theta 0}-R_{\theta 0}^{f}\right)=7.86\times 0.486\times \frac{12300}{373\times 24}\times \left(2.57-0.084\right)=13.04\;\frac{MJ}{m^{3}K}$$

Now we can calculate $N_{1}$:

$$N_{1}=\frac{(23-13.04)\times 361\times 10}{7.86\times 0.486\times (\left(2.57-1.82\right)-0.081)}=14,069\;cycles$$

Therefore, the total life $N_{f}=26,369$. The number of cycles obtained from the experimental test is 26,790, i.e., 1.59% error.

## 7. Summary and Conclusions

The fatigue behavior of Carbon Steel 1018 under sudden changes in frequency is investigated. Fully reversed bending tests are conducted, and the frequency of tests is manipulated with the help of a stepper motor. A model is developed to account for the sudden frequency changes that affect the temperature evolution profile during the fatigue process. It should be noted that this method does not account for sudden shocks in which the specimen’s temperature may not become steady. The findings of the study are summarized below:

1. There is a relationship between the frequency of testing and the life of CS 1018 in the range of testing frequencies. An improvement in fatigue life is observed for higher frequencies compared to lower frequencies, due to the time for damage accumulation in lower frequencies.
2. The material fails when the accumulated generated entropy reaches a nearly constant value of 23 MJm^−3^ K^−1^, with a narrow band range of ±3 MJm^−3^ K^−1^.
3. The results show that, in the tested frequency range, fatigue life improves when the material experiences variable working frequencies. This is attributed to the distribution of stress in variable frequencies as opposed to the concentration of stress at the same frequency for constant frequencies. It should be noted that the results are valid for CS 1018 at the range of tested frequency for both low- and high-cycle fatigue. It is shown that the current model is able to accurately predict the life of a material under rapid frequency variations. However, the material’s fatigue behavior may be subject to change for frequencies at ranges of kHz. Therefore, the application of this method for very-high-cycle fatigue (VHCF) needs further investigations.
4. The life trends also show a dependency on the order of applied frequency in which the life is higher for H–L–H compared to L–H–L. Moreover, for L–H, a higher number of cycles that fail are observed than in H–L tests. Generally, it is discovered that the higher number of changes in frequency (for the tested frequency range for CS 1018) improves life more.
5. Internal friction for 30 Hz and 10 Hz testing frequencies are extracted, and it is shown there is not a significant change in line equations for the tested frequencies. However, a larger slope at 30 Hz is observed due to the higher energy rate given to the system, which activates more half-planes to travel to their neighboring energy levels.

## Figures and Tables

**Table 1:** Chemical composition of tested Carbon Steel 1018.

	Carbon	Manganese	Silicon	Phosphorus	Sulfur	Iron
**CS 1018**	0.13–0.20%	0.30–0.90%	0.15–0.30%	0.04% Max	0.5% Max	98.06–99.42%

**Table 2:** Mechanical properties of tested Carbon Steel 1018.

	UTS (MPa)	YS (MPa)	Modulus of Elasticity (GPa)	Density (g/cm^3^)	Poisson’s Ratio	Hardness, Brinell
**CS 1018**	440	370	205	7.86	0.29	126

**Table 3:** Life of the specimen under 10.16 mm displacement and the life improvement with regards to low-frequency test.

Displacement (mm)	Corresponding Stress Level (MPa)	Frequency Sequencing	Frequency (Hz)	Life (Cycles)	Life Change (% Increase with Respect to Low Frequency)
3.81	211	High	26	489,200	
	211	Low–High	9–26	493,100	0.79
5.08	254	Low	9	46,300	
	254	High	26	46,800	1.07
	254	Low–high	9–26	47,700	3.02
	254	High–low	25–9	47,300	2.15
	254	Low–high–low	8–24–9	48,510	4.77
	254	High–low–high	24–10–24	50,120	8.25
7.62	316	Low	9	14,200	
	316	High	26	14,900	4.9
	316	Low–high	9–26	15,600	9.85
	316	High–low	25–9	15,350	8.09
	316	Low–high–low	8–24–9	15,890	11.90
	316	High–low–high	24–10–24	17,100	20.42
10.16	345	Low	9	5400	
	345	High	26	5700	5.55
	345	Low–high	9–26	6240	15.55
	345	High–low	25–9	6000	11.11
	345	Low–high–low	8–24–9	7100	31.48
	345	High–low–high	24–10–24	8060	49.25

## Data Availability

Data are available upon request.
